# Supplementary material for: Communication and quorum sensing in non-living mimics of eukaryotic cells
Source: Nat Commun. 2018 Nov 28;9:5027. doi: 10.1038/s41467-018-07473-7 (PMC6261949; doi:10.1038/s41467-018-07473-7)
Supplement: Supplementary file 1 — Supplementary Information [file 41467_2018_7473_MOESM1_ESM.pdf]

Supplementary Material

for

Communication and quorum sensing in non-living mimics of eukaryotic cells

Niederholtmeyer et al.

Supplementary Table 1. DNA templates used in this study

**Name**	**Function**	**Description**	**Reference**
240x *tetO* array	Array of 240 TetR binding sites (*tetO*), PRS316-240xtetO (Addgene #44755)	Plasmid, high copy number, Ampicillin	Finney-Manchester et *al*. (2013)[1]
pT7-*tetR*-sfGFP	T7 promoter driven synthesis of C-terminal fusion of sfGFP to TetR repressor (TetR-sfGFP)	Plasmid, pTNT vector (Promega), high copy number, Ampicillin	This work
pT7-*tetR*-mCherry	T7 promoter driven synthesis of TetR-mCherry fusion protein	Plasmid, pTNT vector (Promega), high copy number, Ampicillin	This work
pT3- *tetR*-sfGFP	T7 promoter driven synthesis of TetR-sfGFP	Plasmid, pSB1C3 (Registry of Standard Biological Parts), high copy number, Chloramphenicol	This work
pT7-T3RNAP	T7 promoter driven synthesis of T3 RNA polymerase (only used as PCR template)	Plasmid, Low copy number, pSC101 origin, Kanamycin	This work
pT7-T3RNAP	T7 promoter driven synthesis of T3 RNA polymerase	Linear DNA, PCR amplification of functional region from pT7-T3RNAP plasmid	This work

Supplementary Table 2. DNA content of cell-mimics used in this study. Listed are DNA concentrations used in inner aqueous phase for double emulsion production.

Name and Description	DNA concentrations
tetR-sfGFP / *tetO*<br>Production and capture of TetR-sfGFP	50 nM pT7_*tetR*-sfGFP plasmid<br>20 nM 240x *tetO* array plasmid
tetR-mCherry / *tetO*<br>Production and capture of TetR-mCherry	50 nM pT7_*tetR*-mCherry plasmid<br>20 nM 240x *tetO* array plasmid
Sender cell-mimics<br>Production of TetR-sfGFP	100 nM pT7_*tetR*-sfGFP plasmid
Receiver cell-mimics<br>Capture of TetR-sfGFP	20 nM 240x *tetO* array plasmid
Activator cell-mimics<br>Production of T3 RNAP	40 nM pT7_T3RNAP linear DNA
Reporter cell-mimics<br>T3 promoter controlled production and capture of TetR-sfGFP	30 nM pT3_*tetR*-sfGFP plasmid<br>20 nM 240x *tetO* array plasmid
1-color density sensors cell-mimics<br>Artificial quorum sensing	2.5 nM pT7_T3RNAP linear DNA<br>30 nM pT3_*tetR*-sfGFP plasmid<br>15 nM 240x *tetO* array plasmid
2-color density sensor cell-mimics<br>Artificial quorum sensing with 2-color response	2.5 nM pT7_T3RNAP linear DNA<br>20 nM pT3_*tetR*-sfGFP plasmid<br>20 nM pT7_*tetR*-mCherry plasmid<br>15 nM 240x *tetO* array plasmid

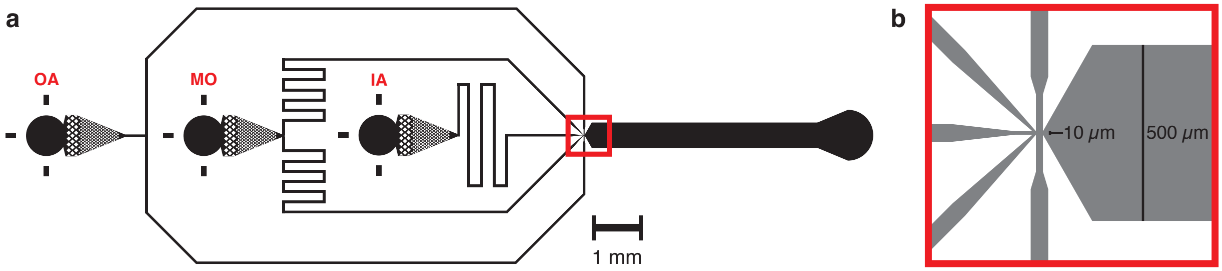

Supplementary Figure 1. Design of the microfluidic device.
a) Design of the microfluidic device used to produce cell-mimics. Inlets for the outer aqueous (OA), middle organic (MO) and inner aqueous phases (IA) are labeled. A magnified view of the flow focusing junction (red box) is shown in b). At the fluidic junction the width of the IA channel is 10 μm.

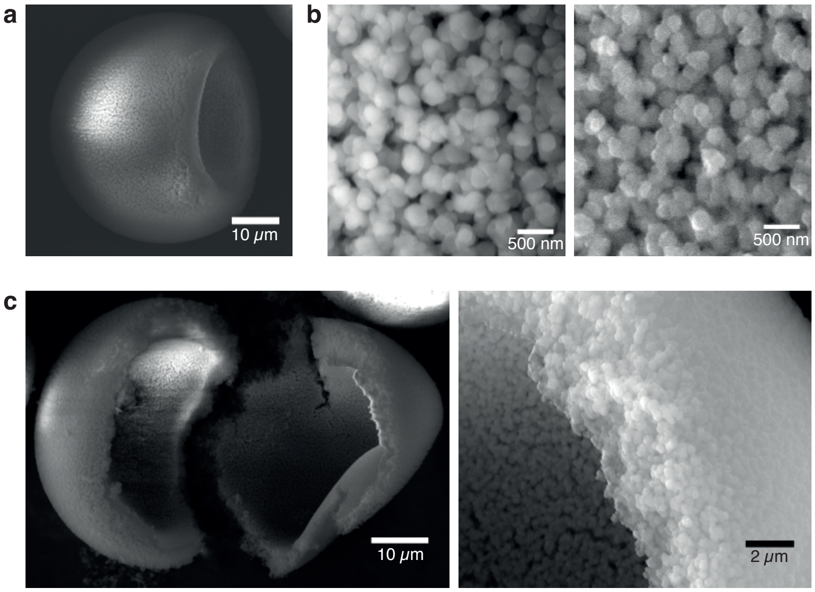

Supplementary Figure 2. Scanning electron microscopy of porous polymer membrane of cell-mimics.
a) Low magnification image of cell-mimic with an indentation. Polymer membranes partly collapsed when cell-mimics dried, which caused indentations in the membrane. b) High magnification images of exterior of microcapsule membranes from two separate batches of cell-mimics. c) Low magnification image of a cracked cell-mimic and magnification of the polymer membrane cross-section.

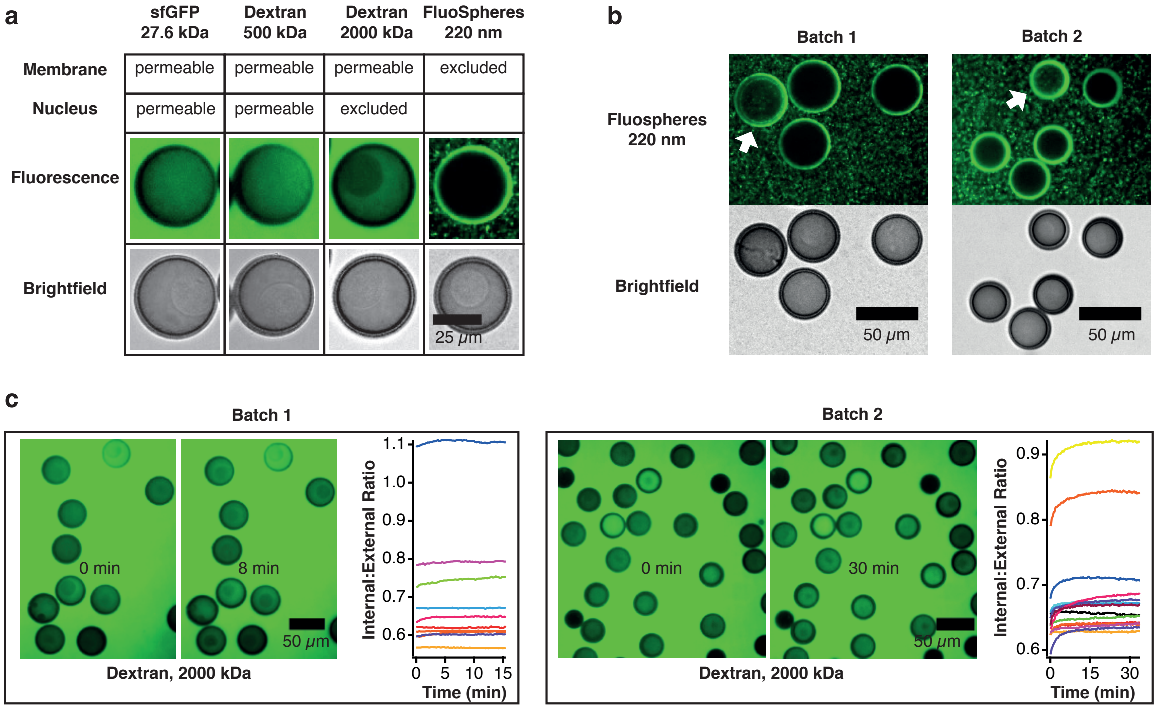

Supplementary Figure 3. Permeability of cell-mimic membranes and clay-DNA hydrogel nuclei.

a) Spinning disk confocal fluorescence images of individual cell-mimics incubated for 24 h with fluorescent tracer molecules of different sizes (top) and corresponding brightfield images (bottom). b) Cell-mimics after incubation with a solution of 220 nm FluoSpheres (Thermo Fisher Scientific). FluoSpheres nanoparticles were excluded from 90% of cell-mimics (n=77, batch 1; n=152, batch 2). Arrows indicate cell-mimics with FluoSpheres in their interior. Many of the cell-mimics with nanoparticles in their interior had visible defects (see brightfield image, batch 1). Images of cell-mimics in a) and b) were acquired after 24 h incubation with labeled tracer molecules in 100 mM HEPES, 0.4% Tween 20. Dextrans were Fluorescein conjugates (Nanocs). c) Diffusion of 2000 kDa dextran into cell-mimics. At time 0, labeled 2000 kDa dextran was added and mixed with cell-mimics in 100 mM HEPES, 0.4% Tween 20. Images were acquired every 15 seconds to monitor diffusion of dextran into the interior of cell-mimics. Fluorescence images show a population of cell- at time 0 and after equilibration of fluorescence signals for cell-mimic batches 1 and 2. Graphs show traces of internal to external fluorescence ratios for individual cell-mimics tracked over time in the shown images. Diffusion of dextran into cell-mimics in batch 2 was slightly slower than for batch 1. An increase in fluorescence could be observed in most batch 2 cell-mimics, while in batch 1, fluorescence levels had already equilibrated at the start of imaging. Final fluorescence intensities varied from cell-mimic to cell-mimic probably because their polymer shells absorbed light to different extends. We observed similar differences in final intensities between cell-mimics for smaller the fluorescent tracer molecules sfGFP and 500 kDa dextran as well. The saturating fluorescence traces show that internal and external 2000 kDa dextran concentrations were equilibrated after 15 min at the latest.

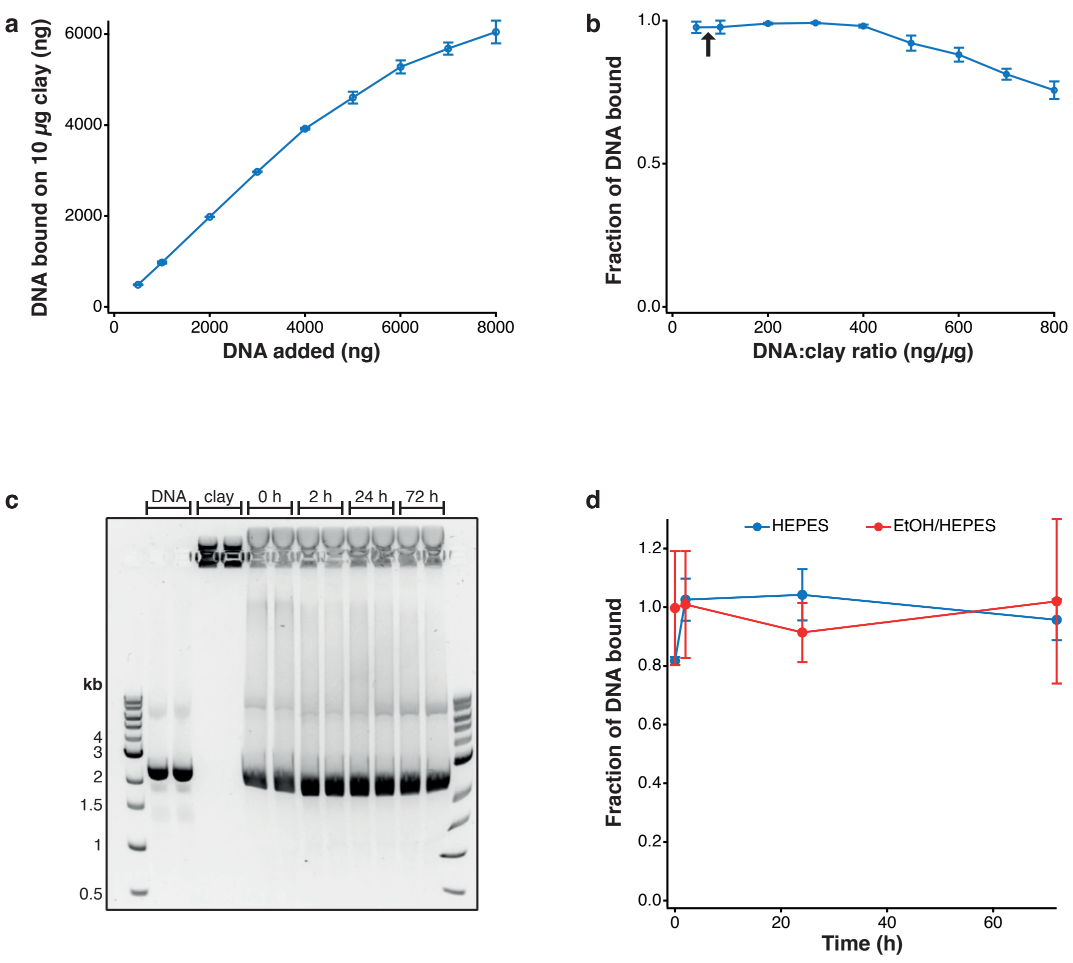

Supplementary Figure 4. Characterization of DNA binding by Laponite XLG clay outside of cell-mimics.

a) Binding curve of plasmid DNA on 10 µg clay. b) Fraction of DNA bound calculated from the same experiment. In cell-mimics, we used DNA at a maximum of 75 ng per µg clay as indicated by the arrow. Binding was measured by titrating plasmid DNA added to 10 µg of Laponite XLG in solution. Hydrogel formation was induced by addition of 200 mM KCl to a final volume of 10 µl. Hydrogel was removed by centrifugation and the DNA left in solution was measured photometrically. Binding experiments were performed in triplicates. Error bars show the standard deviation of the experimental repeats. c, d) Agarose gel analysis of DNA capture and retention under nucleus-formation and cell-mimic storage conditions. Clay-DNA hydrogel was formed at a ratio of 75 ng plasmid DNA per µg clay in 70% ethanol and 200 mM HEPES pH 8. Clay-DNA pellets were stored either in 150 µl 100 mM HEPES pH 8 or in 150 µl 70% ethanol, 200 mM HEPES pH 8 at 4°C for different times to analyze retention of DNA in the hydrogel. Before analysis on an agarose gel, supernatants were removed and hydrogel pellets homogenized. Experiments were performed in duplicate, and each sample was analyzed on two separate gels. c) Example gel for storage in HEPES buffer. The first four lanes were loaded with DNA or clay only. Each lane contained 4 µg clay and 300 ng plasmid DNA. d) DNA amounts in the samples were determined from band intensities on the gel. Error bars are standard deviations of experimental repeats.

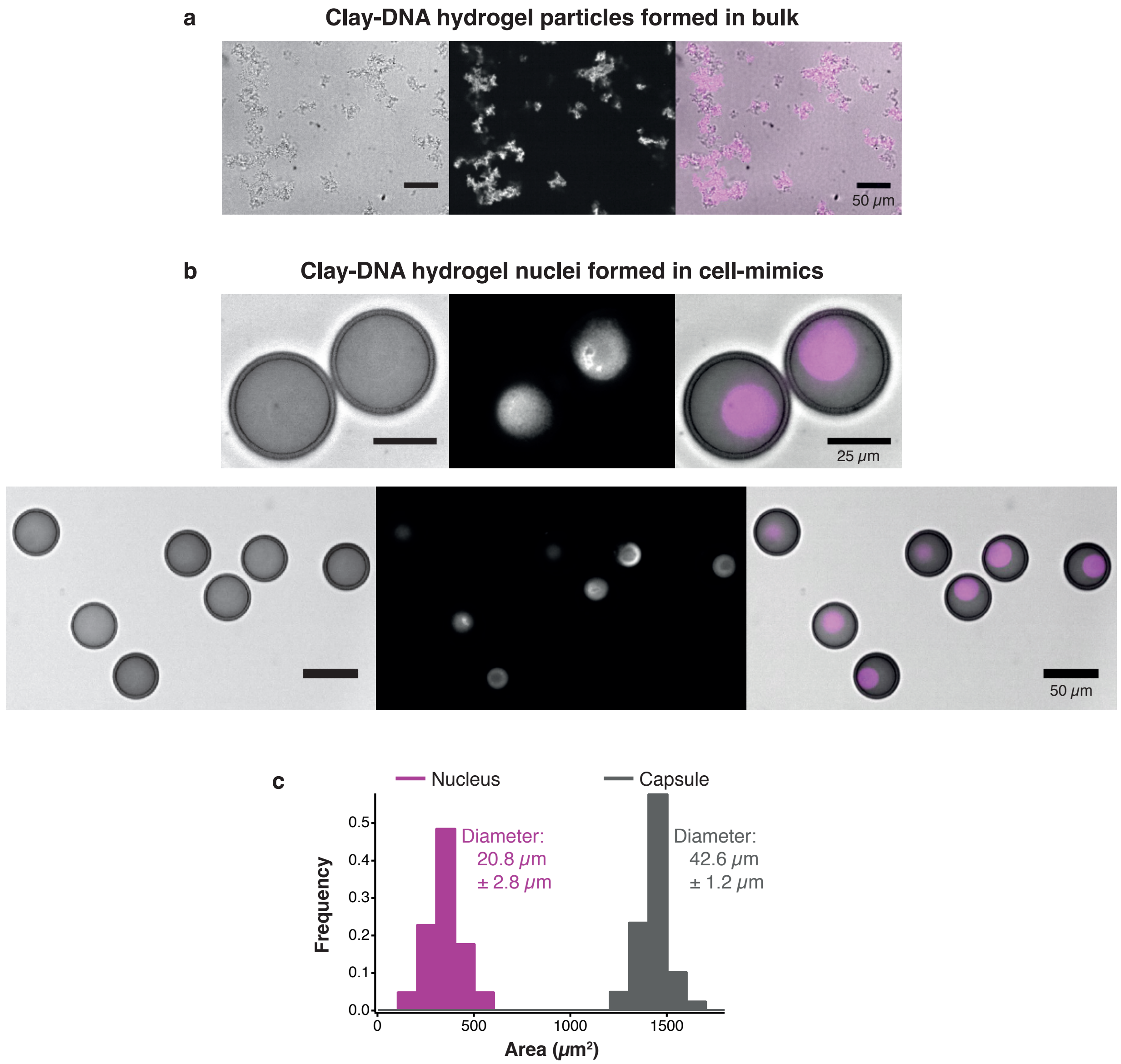

Supplementary Figure 5. Morphologies of clay-DNA hydrogel nuclei.
a) Morphology of clay-DNA hydrogel particles formed in bulk solution. Clay-DNA hydrogel was formed with 300 ng plasmid DNA 4 µg clay in 10 µl volume with 200 mM HEPES pH 8. Hydrogel particles were diluted 1:10 for imaging. Microscopy images show a brightfield image (left), fluorescence of the clay-DNA hydrogel stained with GelRed (center) and a merged image (left). b) Examples of clay-DNA hydrogel nuclei morphologies observed with a 20x (top) and a 63x (bottom) objective. GelRed fluorescence intensities varied in hydrogel nuclei. Intensity of dim hydrogel nuclei did not increase by focusing on a different section in cell-mimics. c) Size distribution of hydrogel nuclei and microcapsules. Areas were measured from GelRed fluorescence and brightfield images for segmented nuclei and microcapsules respectively using Fiji/ImageJ (n = 41). Diameters were calculated from area assuming a circular shape, average and standard deviation are shown. A low percentage (approximately 5 %) of cell-mimics per batch did not contain one defined hydrogel nucleus but fragmented particles.

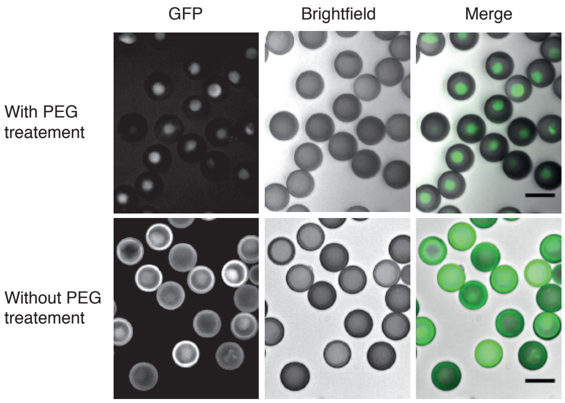

Supplementary Figure 6. PEG treatment prevents non-specific binding of proteins to polymer membranes of cell-mimics.
Endpoint fluorescence of *tetR*-sfGFP / *tetO* cell-mimics (Fig. 1c) and localization of TetR-sfGFP after 3 h TX-TL with or without PEG treatment.

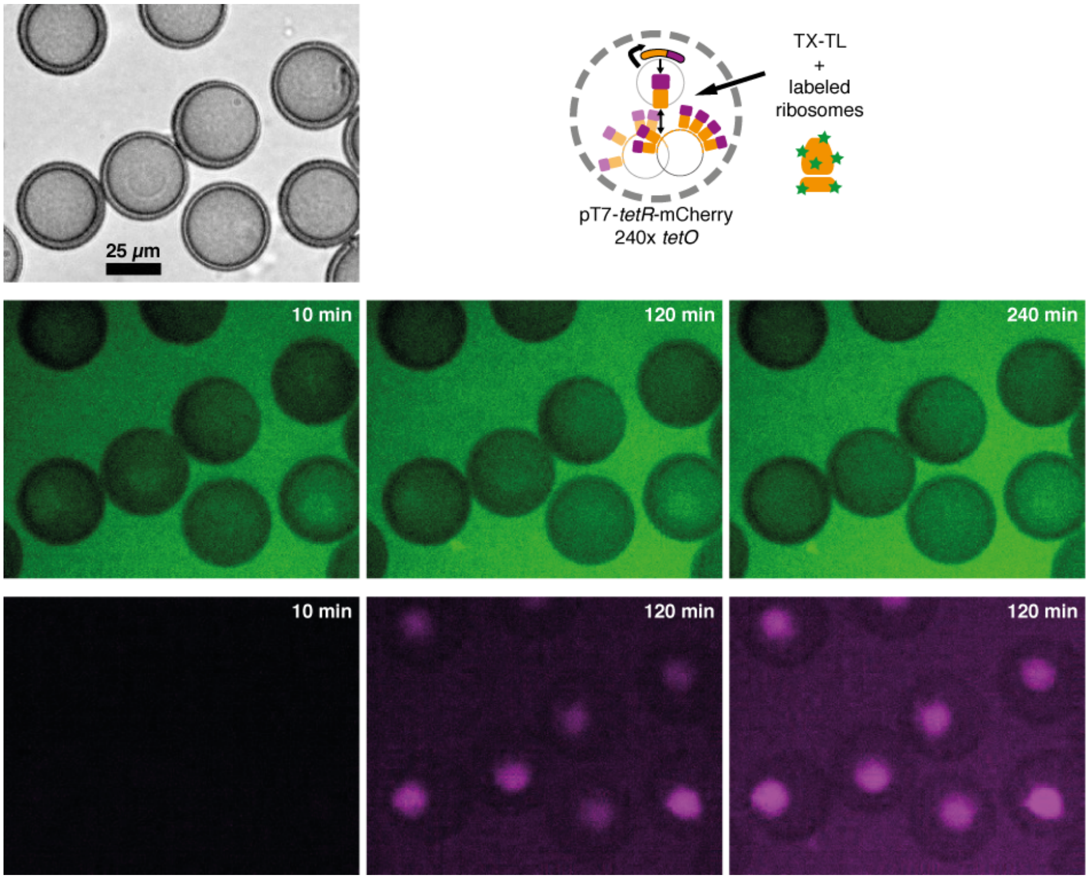

Supplementary Figure 7. Cell-mimics' porous polymer membranes are permeable to ribosomes.
Protein expression in *tetR*-mCherry / *tetO* cell-mimics was initiated by addition of TX-TL reagents spiked with Alexa Fluor 488 labeled *E. coli* ribosomes. Top shows a brightfield image and a schematic of the experiment. Alexa Fluor 488 labeled ribosomes (middle, green) could be detected in cell-mimics interior and in many cell-mimics their concentration was slightly increased in hydrogel nuclei over the rest of the interior. Hydrogel nuclei accumulated TetR-mCherry fluorescence over time (bottom, magenta).

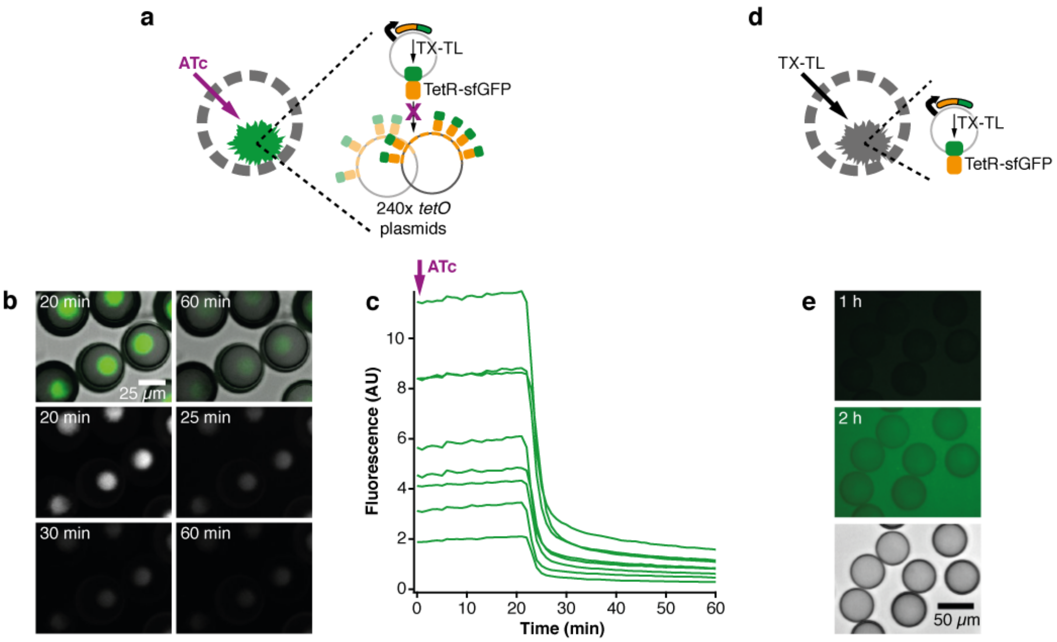

Supplementary Figure 8. Specificity of TetR-sfGFP binding to *tetO* sites in hydrogel nuclei. a-c) Anhydrotetracycline (ATc) induced unbinding from hydrogel nuclei containing 240x *tetO* plasmid. a) Schematic of the experiment. TetR-sfGFP was expressed in cell-mimics in TX-TL as in Fig. 1d. When expression ended after 3 h, cell-mimics were transferred into 200 mM HEPES pH 8, 2 mg/ml BSA. To observe ATc induced unbinding from nuclei, cells in 18 µl of buffer were pipetted into a cylindrical reaction chamber prepared by punching a 2 mm hole into a 10 mm high block of PDMS, which was placed on cover glass for imaging. ATc for a final concentration of 2.5 µM was pipetted on top of the liquid without active mixing. Imaging was started immediately and ATc was left to diffuse to cell-mimics. b) Timelapse images of TetR-sfGFP unbinding. Shown are merged images before and after unbinding (top) and GFP fluorescence images of the fast disappearance of TetR-sfGFP from hydrogel nuclei. c) Dynamics of the unbinding process observed by tracking fluorescence in eight representative hydrogel nuclei over time. d) Schematic of control experiment of *tetR*-sfGFP expression in cells without 240x *tetO* array plasmid. e) Timelapse images of GFP fluorescence (top, middle). To show increase of fluorescence in solution, images were set to a higher brightness than images in panel b. Bottom: brightfield channel.

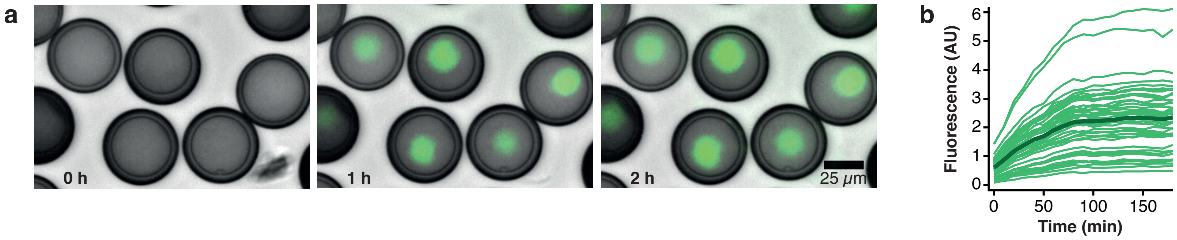

Supplementary Figure 9. Low batch-to-batch variability of protein expression in cell-mimics.
Expression and capture of TetR-sfGFP in a batch of cell-mimics prepared approximately a year later than those shown in Fig. 1. Timelapse images of TetR-sfGFP fluorescence increase in nuclei (green fluorescence merged with brightfield) and b) traces of fluorescence increase in 40 nuclei and averaged fluorescence (bold line).

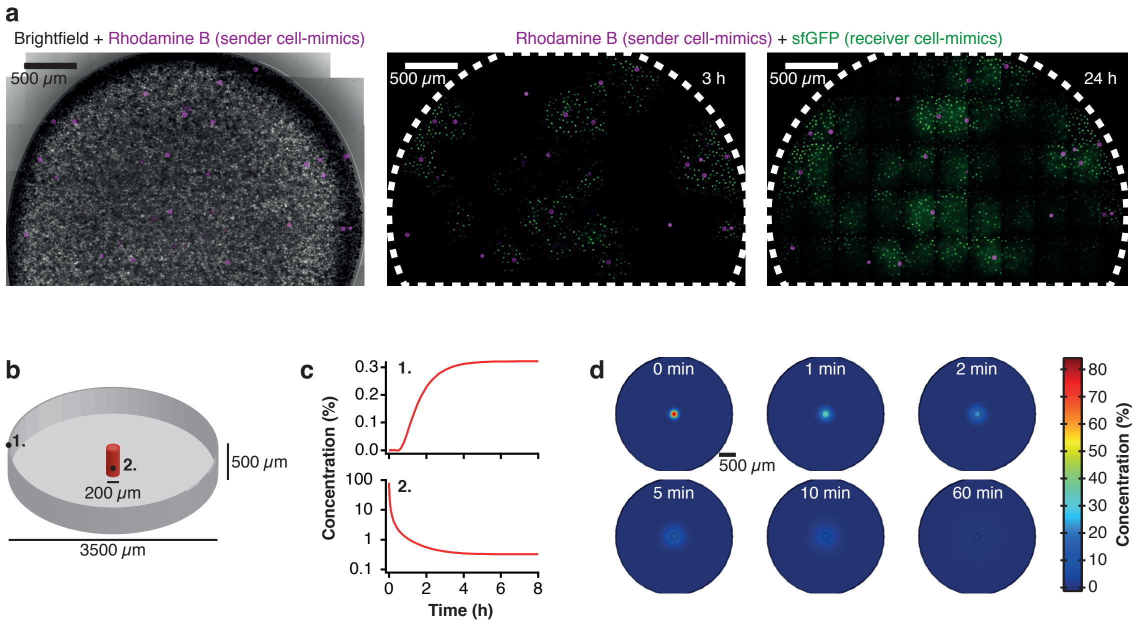

Supplementary Figure 10. Protein exchange in a large droplet of sender and receiver cell-mimics.

a) Brightfield image of dense sender and receiver cell-mimic colony shown in Fig. 2b, merged with fluorescence form sender cell-mimics (magenta). Distribution of TetR-sfGFP fluorescence (green) is shown after 3 h and 24 h with identical brightness settings. b-d) COMSOL simulation of free diffusion of TetR-sfGFP (50.1kDa) in a geometry comparable to the experiment in a). We assumed a diffusion coefficient of $6*10^{-7}$ cm^2 s^{-1}, which was measured for 67 kDa bovine serum albumin[2]. b) Schematic and dimensions of the geometry used in the simulation. Protein was initially located in a cylindrical region (red) in the center. Protein could freely diffuse in the entire grey cylinder, which was closed on the top and bottom. c) Concentration change in the two positions indicated in b). d) Surface concentration across the geometry at different time points.

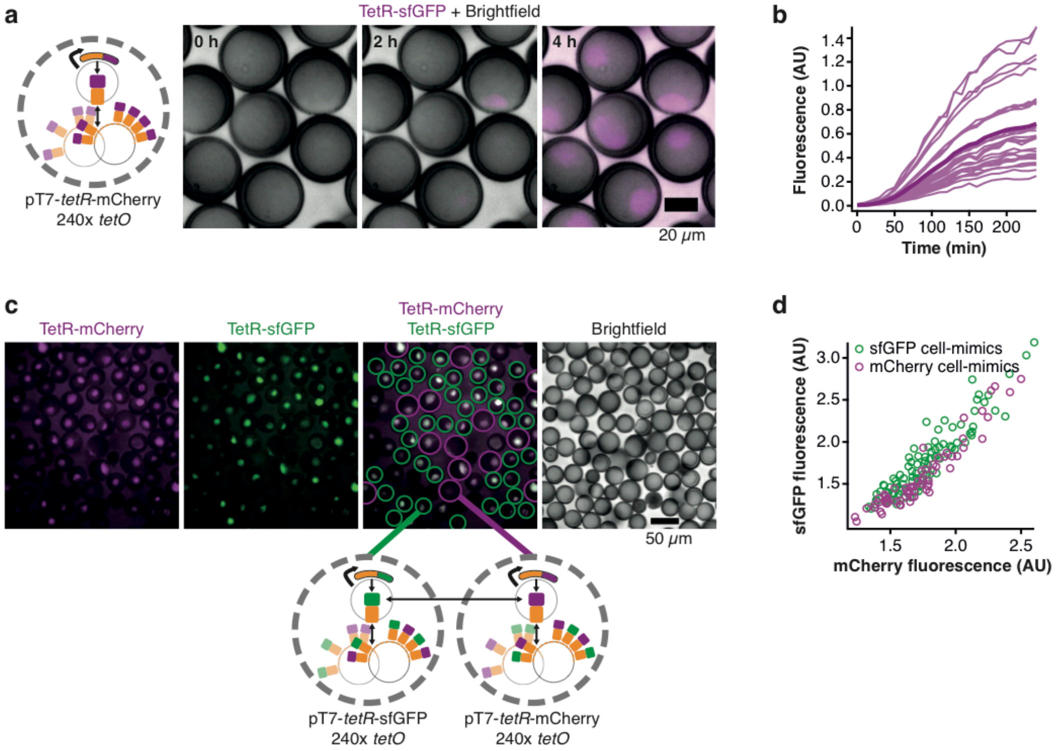

Supplementary Figure 11. Protein exchange between TetR-mCherry and TetR-sfGFP producers.

a) Schematic of cell-mimics expressing and binding TetR-mCherry (*tetR*-mCherry / *tetO*) and time lapse images of TetR-mCherry fluorescence (magenta) merged with brightfield images. b) Traces of fluorescence increase in hydrogel nuclei of 27 *tetR*-mCherry / *tetO* cell-mimics with the average in bold. c) Endpoint fluorescence (4 h) in a mix of *tetR*-sfGFP / *tetO* and *tetR*-mCherry / *tetO* cell-mimics. As shown in the schematic below, both cell-mimics types contain the 240x *tetO* plasmid and bind a mix of both fluorescent reporter proteins. Images show fluorescence channels separately and a merge of both fluorescence channels with circles indicating the positions of TetR-sfGFP producers (green) and TetR-mCherry producers (magenta), which were identified from the brightfield image by their difference in size and cell wall thickness. d) Correlation of sfGFP and mCherry fluorescence in TetR-sfGFP producers (green) and TetR-mCherry producers (magenta). Each data point shows sfGFP and mCherry fluorescence in the hydrogel nucleus of an individual cell-mimic. The cell-mimic types cannot be distinguished from each other by their respective fluorescence levels. Cell-mimics that could not be classified into a category based on their appearance in the brightfield channel were not analyzed.

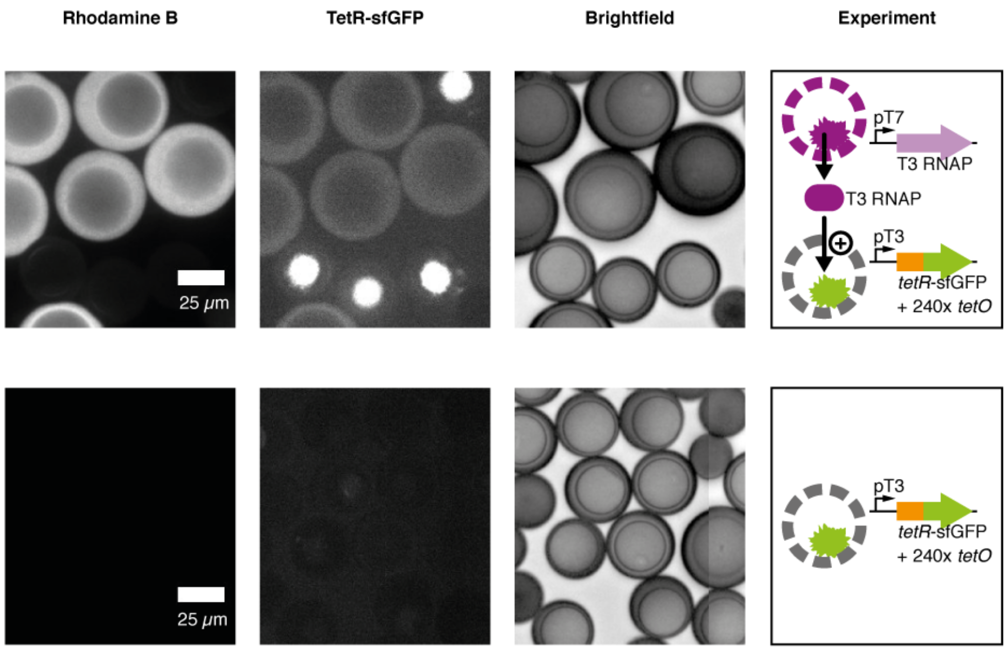

Supplementary Figure 12. Activation of reporter cell-mimics by T3 RNAP producing activator cell-mimics and control experiment.
Images of endpoint fluorescence (3 h) and brightfield channel from activation experiment with a mix of activator and reporter cell-mimics as well as a control experiment using reporter cell-mimics only as shown in the schematic. Activator cell-mimics were labeled with Rhodamine B in their polymer membranes.

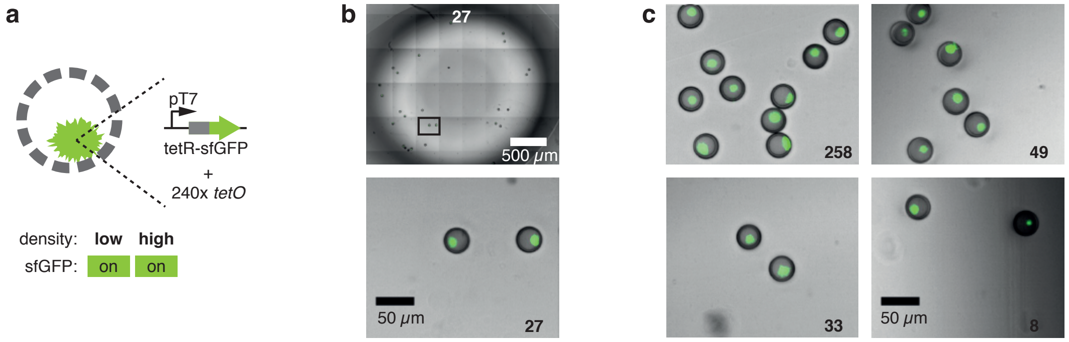

Supplementary Figure 13. Constitutive producers of TetR-sfGFP show the reporter protein in their hydrogel nuclei at low cell-mimic densities.

a) Schematic of control cell-mimics for the artificial quorum sensing experiments (Fig. 4) that express the reporter protein under control of a constitutive T7 promoter and contain the 240x *tetO* plasmid. Independent of cell density, control cells accumulated reporter protein in their nuclei. Constitutively expressing *tetR*-sfGFP / *tetO* control cell-mimics cell-mimics were as in Fig. 1C-D. b) Experiments were performed like in Fig. 4 in droplets of 4.5 µl TX-TL containing different numbers of control cell-mimics as indicated. Lower image is a magnification of the region highlighted above. c) Magnifications of representative control cells in droplets containing the indicated number of cells. All images were set to identical brightness settings.

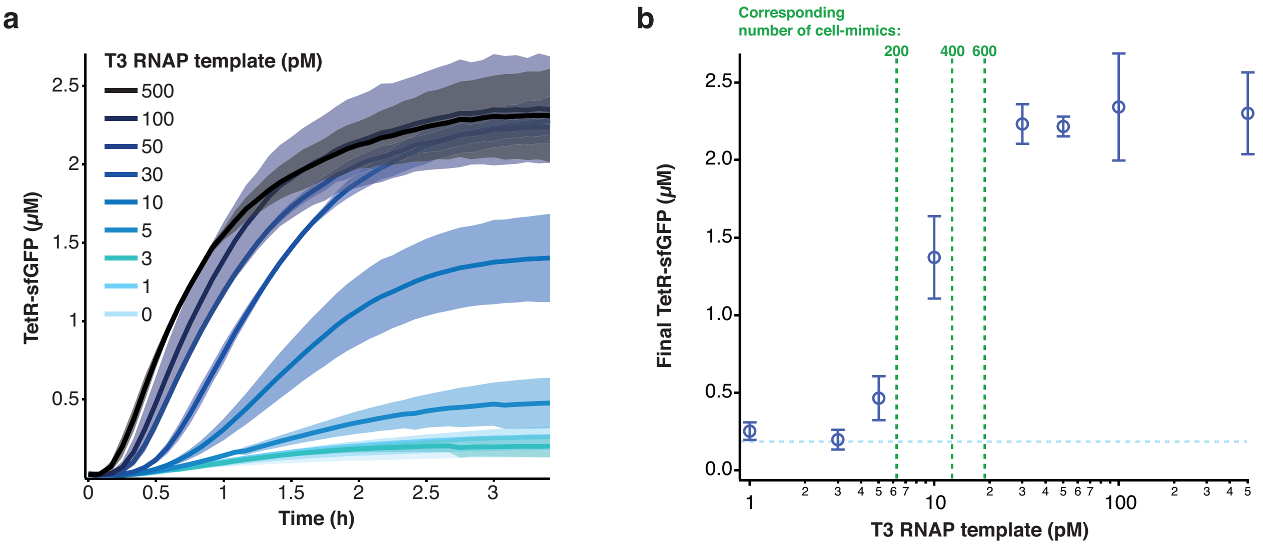

Supplementary Figure 14. Titration of T3 RNAP template DNA in batch reactions.
a) Dynamics of TetR-sfGFP production from a T3 promoter in TX-TL reactions containing different amounts of T3 RNAP template DNA. GFP fluorescence was read on a platereader in reactions containing 5 nM pT3-tetR-sfGFP reporter plasmid and variable amounts of pT7-T3RNAP linear template DNA. All reactions were done at least in triplicate and shaded areas indicate standard deviations between experimental repeats. b) Final TetR-sfGFP produced in a) as a function of T3 RNAP template concentration. Light blue dashed line indicates fluorescence of a reaction with no T3 RNAP template. Green dashed lines indicate calculated T3 RNAP template concentrations in a 4.5 µl droplet containing 200, 400 and 600 artificial quorum sensing cell-mimics. 400 cell-mimics per 4.5 µl was the lowest density at which expression of the reporter was observed in Fig. 4c.

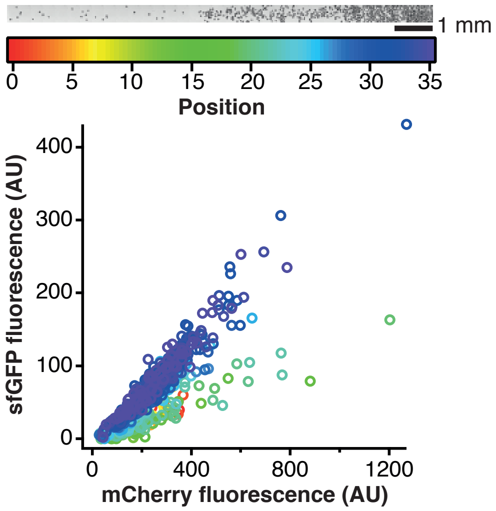

Supplementary Figure 15. Crowded and dilute 2-color density sensors display sfGFP to mCherry fluorescence in two distinct populations.
Fluorescence of individual cell-mimics in the density gradient experiment shown in Fig. 4e is plotted as sfGFP versus mCherry fluorescence. Positions of individual cell-mimics in the gradient are color coded as shown in the color legend and the corresponding image above.

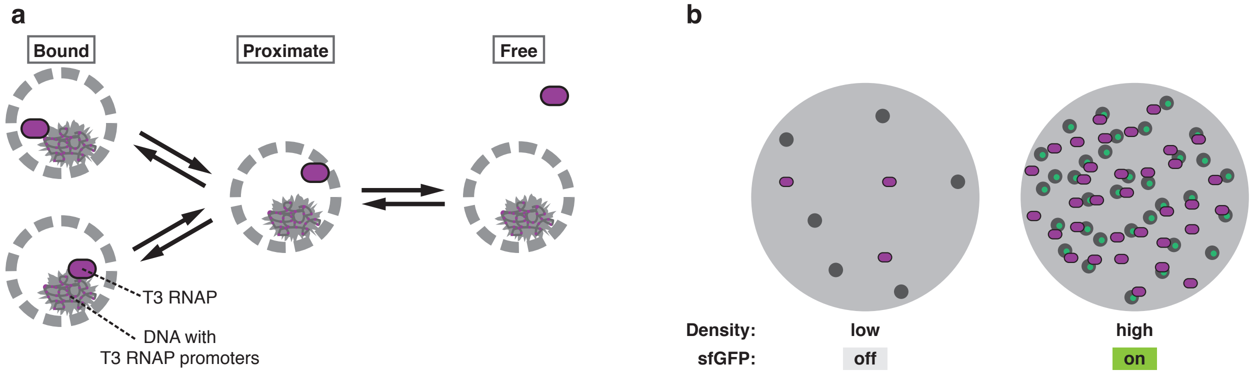

Supplementary Figure 16. Proposed mechanism for switch-like response of gene expression to cell-mimic density.

a) Binding of T3 RNAP to polyvalent hydrogel nuclei of cell-mimics. Hydrogel nuclei of artificial quorum sensing cell-mimics contain high local concentrations of T3 RNA polymerase (T3 RNAP) promoters, estimated to be approximately 200 nM in hydrogel nuclei considering their condensed size with respect to the cell-mimics' volume, which corresponds to approximately 200'000 T3 RNAP promoters per nucleus. Because hydrogel nuclei are highly polyvalent, once a T3 RNAP is bound to a nucleus it is very likely to rebind the same nucleus after briefly dissociating from its DNA. Model adapted from Klein et al. 2003[3]. b) Schematic of cell-mimic and T3 RNAP distributions at low and high cell-mimic densities. At low cell-mimic densities, T3 RNAP is at low concentration and distances between cell-mimics are large. The chances for a free T3 RNAP to encounter a T3 RNAP promoter inside a cell-mimic's nucleus are therefore low. At higher cell-mimic densities T3 RNAP concentrations are higher, because more cell-mimics are present to produce T3 RNAP, and at the same time, distances between cell-mimics are smaller, so that chances are higher for binding a nucleus. Then, once bound, a T3 RNAP molecule, likely stays associated with the same hydrogel nucleus due to repeated rebinding and keeps producing reporter mRNA until expression ends.

Supplementary References

1. Finney-Manchester, S. P. & Maheshri, N. Harnessing mutagenic homologous recombination for targeted mutagenesis in vivo by TaGTEAM. *Nucleic Acids Res.* **41,** e99–e99 (2013).
2. Tyn, M. T. & Gusek, T. W. Prediction of diffusion coefficients of proteins. *Biotechnology and bioengineering* **35,** 327–338 (1990).
3. Klein, P., Pawson, T. & Tyers, M. Mathematical Modeling Suggests Cooperative Interactions between a Disordered Polyvalent Ligand and a Single Receptor Site. *Current Biology* **13,** 1669–1678 (2003).
