# Supplementary material for: Communication and quorum sensing in non-living mimics of eukaryotic cells
Source: Nat Commun. 2018 Nov 28;9:5027. doi: 10.1038/s41467-018-07473-7 (PMC6261949; doi:10.1038/s41467-018-07473-7)
Supplement: Supplementary file 6 — Description of Additional Supplementary Files [file 41467_2018_7473_MOESM6_ESM.docx]

**Title:** Movie 1.
**Description:** Production of double emulsion droplets. Timelapse movie of the formation of water-in-oil-in-water emulsion droplets for the production of artificial cellmimics with hydrogel nuclei. Some inhomogeneity can be observed in droplet sizes and breakage of droplets that results in polymer beads after polymerization. Flow rates were 300 µl/h for the outer aqueous, 40 µl/h for the middle organic and 12 µl/h for the inner aqueous phase.

**Title:** Movie 2.
**Description:** Expression and capture of TetR-sfGFP in hydrogel nuclei of cellmimics. Timelapse movie of the expression of TetR-sfGFP in cell-mimics containing pT7-tetR-sfGFP expression and 240x tetO array plasmids (see Fig. 1). Merge of brightfield channel and sfGFP fluorescence (green).

**Title:** Movie 3.
**Description:** Communication between cell-mimics via a diffusive genetic activator signal. Activation of gene expression from T3 RNAP producing activator cellmimics (magenta) to reporter cell-mimics (see Fig. 3). Merge of brightfield channel, sfGFP fluorescence (green) and rhodamine B fluorescence (magenta).
